# A working model for the formation of Robertsonian chromosomes

**DOI:** 10.1242/jcs.261912

**Published:** 2024-04-12

**Authors:** Jennifer L. Gerton

**Affiliations:** Stowers Institute for Medical Research, Kansas City, MO 64110, USA

**Keywords:** Robertsonian, Chromosomes, Meiosis, Recombination, Translocation, Karyotype

## Abstract

Robertsonian chromosomes form by fusion of two chromosomes that have centromeres located near their ends, known as acrocentric or telocentric chromosomes. This fusion creates a new metacentric chromosome and is a major mechanism of karyotype evolution and speciation. Robertsonian chromosomes are common in nature and were first described in grasshoppers by the zoologist W. R. B. Robertson more than 100 years ago. They have since been observed in many species, including catfish, sheep, butterflies, bats, bovids, rodents and humans, and are the most common chromosomal change in mammals. Robertsonian translocations are particularly rampant in the house mouse, *Mus musculus domesticus,* where they exhibit meiotic drive and create reproductive isolation. Recent progress has been made in understanding how Robertsonian chromosomes form in the human genome, highlighting some of the fundamental principles of how and why these types of fusion events occur so frequently. Consequences of these fusions include infertility and Down's syndrome. In this Hypothesis, I postulate that the conditions that allow these fusions to form are threefold: (1) sequence homology on non-homologous chromosomes, often in the form of repetitive DNA; (2) recombination initiation during meiosis; and (3) physical proximity of the homologous sequences in three-dimensional space. This Hypothesis highlights the latest progress in understanding human Robertsonian translocations within the context of the broader literature on Robertsonian chromosomes.

## Teaser

**Summary:** Robertsonian chromosomes are common in nature and can lead to reproductive isolation and speciation. Recent technological advances have led to new insights into how they form in the human genome.

## Definition of a Robertsonian chromosome

The primary constrictions on mitotic chromosomes are usually centromeres, which were first described by Walther Flemming (Flemming, 1882). Centromeres can be located either in the middle region of a chromosome, termed metacentric, or near the end of a chromosome, termed telocentric if abutting the telomere or acrocentric if there is a short arm between the centromere and telomere (see Glossary). Robertsonian chromosomes (ROBs, also abbreviated as Rbs in mice) result from translocations or fusions between two telocentric or acrocentric chromosomes that create a single metacentric chromosome.

**Glossary**

**Acrocentric:** the morphology of a chromosome that has a short region between the telomere and centromere; the centromere is positioned asymmetrically relative to the entire chromosome.

**Alternate segregation outcome:** a balanced meiotic segregation in the carrier of a ROB.

**Extrachromosomal DNA:** DNA that is found variably in a karyotype and not in a chromosome.

**Fluorescence *in situ* hybridization:** a process in which a fluorescently labeled DNA sequence is hybridized to chromosomes, followed by visualization by microscopy, to demonstrate the position of the DNA sequence.

**Haplotype:** a region of DNA that tends to be inherited as a block.

**HiC:** a method to detect genome-wide chromatin interactions that combines conformation capture by crosslinking with next-generation DNA sequencing.

**Interchromosomal effect:** the influence of an atypical chromosome on the segregation of other unaffected chromosomes.

**Karyomorphs:** distinct karyotypes associated with a single species.

**Karyotype:** the chromosomes that make up the genome of a species.

**Kinetochore:** a proteinaceous structure that forms at centromeres and attaches to microtubules, enabling sister chromatid segregation.

**Major satellite DNA:** satellite DNA that is present on all mouse chromosomes and has centromeric cohesion function.

**Meiotic drive:** inheritance of genetic material in a non-Mendelian ratio.

**Metacentric:** the morphology of a chromosome that has the centromere positioned near the middle.

**Minor satellite DNA:** satellite DNA that is present on all mouse chromosomes and has kinetochore function.

**Non-allelic homologous recombination:** recombination that relies on sequence similarity occurring outside of the matched allelic position on the homologous chromosome.

**Polar bodies:** non-gametic bodies formed during female meiosis, containing either half of the material segregated during meiosis I or half of the material segregated during meiosis II.

**Pseudoautosomal regions:** regions of near identity between X and Y chromosomes that crossover in meiosis.

**Pseudohomolog regions:** DNA sequences of near identity between human acrocentric chromosomes, found on the p arms.

**Reproductive isolation:** the inability of two individuals or subpopulations of the same species to produce offspring, which can be due to incompatibilities in karyotype.

**Sexual dimorphism:** a feature that is systematically distinct between the two sexes of a species.

**Sister chromatid cohesion:** the force that holds sister chromatids together and enables sister chromatids to biorient on the mitotic spindle.

**Subfertility:** reproductive capacity that is lower than that characteristic for a species.

**Synaptonemal complex:** a proteinaceous structure that forms between a pair of homologous chromosomes in meiosis.

**Tandem repeat arrays:** a DNA sequence that is repeated consecutively in a head-to-tail arrangement.

**Telocentric:** the morphology of a chromosome that has a telomere immediately abutting a centromere.

**Telomeric fusion:** the fusion of two chromosomes at their ends.

## Robertsonian translocations are rampant in nature

**Fig. 1.**
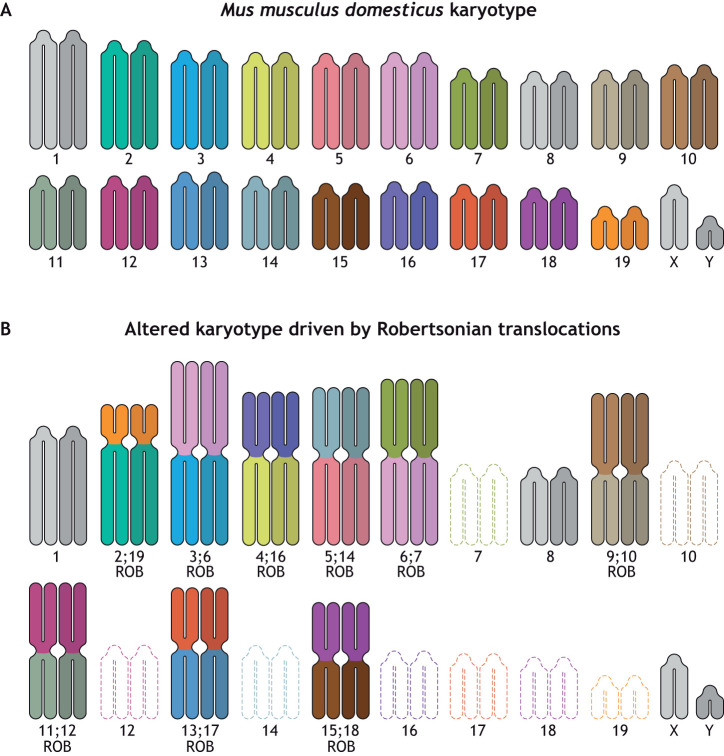
**Karyotypes of *Mus musculus domesticus*.** (A) The laboratory strain of *M. m. domesticus* has a karyotype of 20 pairs of telocentric chromosomes, in which the centromeres are immediately adjacent to telomeres. Centromeres of all the chromosomes are composed of two types of satellite DNA: minor satellite DNA, where the kinetochore assembles; and major satellite DNA, where the site of sister chromatid cohesion is located. (B) A karyotype is shown from a *M. m. domesticus* individual from the small volcanic island of Madeira (Britton-Davidian et al., 2000). This karyotype has 11 pairs of metacentric chromosomes that are the result of fusions of the chromosomes indicated. This karyotype is one of six distinct chromosomal ‘races’ documented on Madeira, which are separated from each other by mountain barriers.

A karyotype is the number and characteristic shape of the chromosomes of a species. For example, a typical inbred laboratory mouse (*Mus musculus*) karyotype consists of 20 pairs of telocentric chromosomes (Fig. 1A). Telocentric chromosomes do not exist in the human karyotype, but five of the 23 pairs of human chromosomes are acrocentric. Although we often think of a karyotype as a fixed characteristic of the genome of a species, we also recognize that genomes can be malleable, as exemplified by the inclusion of plasmids in bacteria or extrachromosomal DNA in eukaryotes (see Glossary). Chromosomes and karyotypes can be artificially altered, especially with the advent of CRISPR-Cas9 technology: organisms with CRISPR-Cas9-engineered chromosomal fusions have been made successfully in budding yeast (Luo et al., 2018; Shao et al., 2018), fission yeast (Gu et al., 2022) and mice (Zhang et al., 2022). Fusion events can also occur naturally and have been recognized for over 100 years. Robertson first discovered the fusion of two chromosomes to make a metacentric chromosome in a karyotype of a grasshopper, hence the name Robertsonian chromosome (Robertson, 1916). Robertsonian fusions are the most common chromosomal change in mammals (Qumsiyeh, 1994), and ROBs have been detected in most mammalian lineages (Ferguson-Smith and Trifonov, 2007). ROBs occur naturally in many species *de novo*, as has been documented in butterflies (Lukhtanov et al., 2005), faba bean (Schubert et al., 1992), catfish (Glugoski et al., 2023), cattle (Blazak and Eldridge, 1977; Ducos et al., 2008; Gustavsson, 1979; Holeckova et al., 2021) and mice (Garagna et al., 2014), among others. The results of several large-scale cytogenetic studies suggest that the incidence of ROBs in the human population is 1 in 800 (Hamerton et al., 1975; Nielsen and Wohlert, 1991; Poot and Hochstenbach, 2021; Zhao et al., 2015). Approximately half of human ROBs are inherited and half form *de novo* (Hook and Cross, 1987).

There are many examples of chromosomal fusions that distinguish the genomes of closely related species, a situation sometimes referred to as chromosomal speciation (Brannan et al., 2023; Ferguson-Smith and Trifonov, 2007). ROBs create challenges for pairing to native chromosomes in meiosis (Luciani et al., 1984; Vidal et al., 1982). From an evolutionary and population genetics perspective, when meiosis is unsuccessful in a particular mating context, the consequences are subfertility, reproductive isolation and, potentially, speciation (see Glossary) (Brannan et al., 2023; Ferguson-Smith and Trifonov, 2007). One suspected example of chromosomal speciation is the fusion of chromosomes 2A and 2B after the split between chimpanzees and humans, yielding what is chromosome 2 in the human genome today (Yunis and Prakash, 1982), although this example is a case of telomeric fusion (see Glossary) rather than a classic Robertsonian fusion. This particular fusion event, now fixed, was coincident with a major bottleneck in human ancestry (Hu et al., 2023), suggesting that it might have been a significant contributing factor. The high frequency of *de novo* ROBs is an economic burden to the livestock breeding industry, including cattle, sheep and pig, since it manifests as subfertility, small litter sizes, infertility, aborted fetuses and stillborn offspring (Lewis et al., 2022; O'Connor et al., 2021). The Equidae family, which includes horses, donkeys and zebras, diverged from a common ancestor of tapirs and rhinoceroses around 4 million years ago, and this event was accompanied by a shift from predominantly acrocentric chromosomes to fewer mainly metacentric chromosomes (Trifonov et al., 2012, 2008), which is consistent with chromosomal speciation via formation of ROBs. The incidence and impact of ROBs on reproduction has been intensively studied in the experimentally tractable house mouse, yielding significant insight into their consequences.

## Robertsonian chromosomes in the house mouse

Chromosomes from wild populations of the house mouse, *Mus musculus domesticus*, are notable for their extraordinary propensity to form ROBs. Since 1969, scientists have documented many geographical ‘races’ of the house mouse spread across Europe and Africa, with nearly 100 different documented karyomorphs (see Glossary) (Garagna et al., 2014). The standard karyotype of the laboratory mouse consists of 20 pairs of telocentric chromosomes (Fig. 1A). However, in the wild, every one of these chromosomes has been found fused to every other chromosome, with the exception of the Y chromosome. On the small volcanic island of Madeira alone, six distinct karyomorphs have been documented (Fig. 1B) (Britton-Davidian et al., 2000). The structure of naturally occurring ROBs suggests that the breakpoint (the position and sequence where two chromosomes are joined to make a single chromosome) lies in the centromeric satellite DNA (Garagna et al., 1995; Nanda et al., 1995). Once a metacentric chromosome is formed, one of the arms can be swapped for a different telocentric chromosome, referred to as whole-arm reciprocal translocation (WART) (Capanna and Redi, 1995), a process that also occurs in ROBs in other species (Mao et al., 2008). The telocentric chromosomes in mice can be induced to form end-to-end fusions in the laboratory, recognized as ROBs, if the telomeres are critically short, as occurs in the telomerase-mutant mouse (Blasco et al., 1997). Thus, ROBs can form by different mechanisms and therefore have different structures. Regardless, these karyomorphs can create reproductive barriers.

There are additional consequences of ROBs in mice: the Lampson lab has crossed mice bearing the all-telocentric karyotype with mice bearing several metacentric ROBs and studied the transmission of metacentric versus telocentric chromosomes into oocytes during female reproduction. In a series of elegant studies, they demonstrated that the strength of the centromere can drive chromosomes into the oocyte at higher-than-Mendelian ratios through female meiosis (Akera et al., 2019; Chmatal et al., 2014; Kumon et al., 2021). Chromosomes with relatively weaker centromeres end up in ‘dead-end’ polar bodies that form during meiotic divisions (see Glossary). Depending on the ‘matchup’ in the cross between subpopulations with naturally occurring ROBs, the telocentric chromosomes or ROBs may be preferentially retained in the oocyte. Centromere strength is one mechanism of meiotic drive (see Glossary) (Zanders and Unckless, 2019). Meiotic drive has also been documented for ROBs in human female meiosis but not male meiosis (Pardo-Manuel de Villena and Sapienza, 2001b). The asymmetric divisions in female meiosis permits drive via centromere strength (Pardo-Manuel de Villena and Sapienza, 2001a), although additional mechanisms may emerge as we begin to understand the unique structural features of individual ROBs.

## The impact of Robertsonian chromosomes on human health

**Fig. 2.**
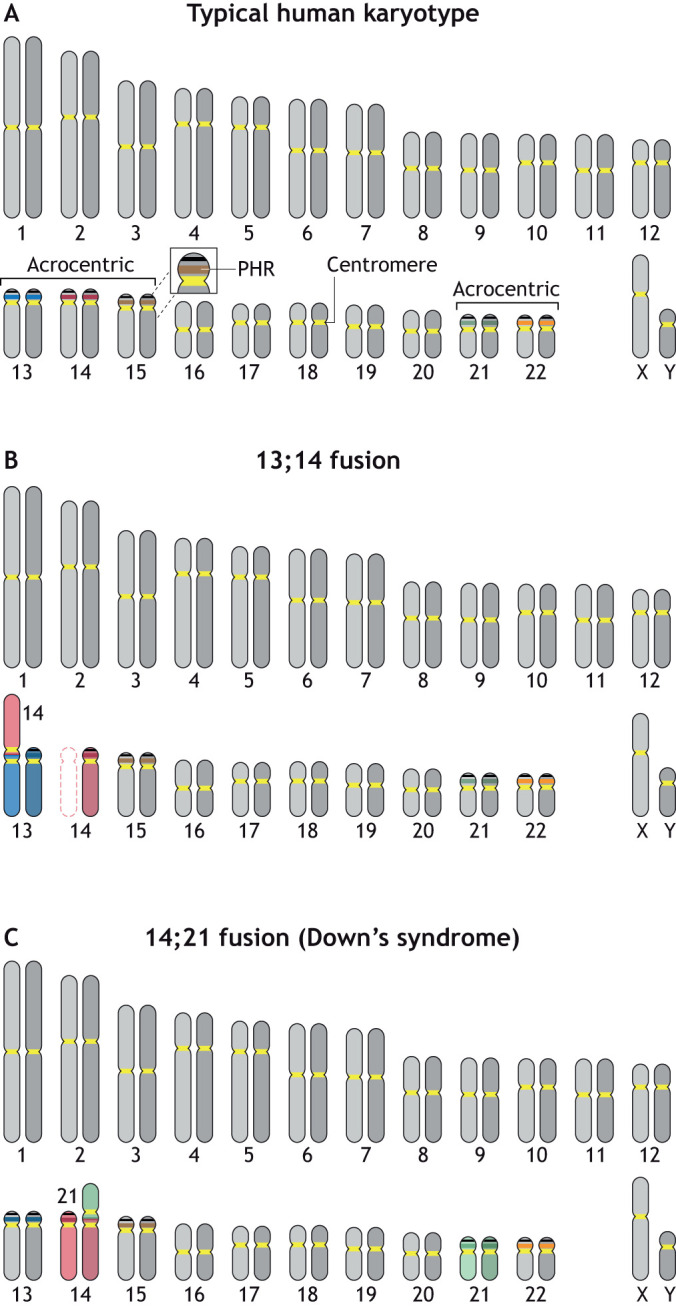
**Human karyotypes.** (A) A typical human karyotype with 23 pairs of chromosomes is shown. Chromosomes 13, 14, 15, 21 and 22 are acrocentric, with a short p arm between the centromere (yellow) and the telomere. These chromosomes share regions of striking homology on their p arms, termed PHRs, indicated by the darker color bands. PHRs range in size from 0.7 Mb to 6.5 Mb in the CHM13 genome. Black bands indicate ribosomal DNA arrays. (B) Karyotype depicting a fusion between chromosomes 13 and 14, the most common ROB event in humans. (C) Karyotype depicting a fusion between chromosomes 14 and 21, along with two normal copies of chromosome 21. This karyotype has a normal number of chromosomes but is effectively trisomic for chromosome 21, representing the karyotype of an individual with Down's syndrome.

The human genome has 23 pairs of chromosomes, including five acrocentric chromosomes with the potential to form ROBs (Fig. 2A). However, only three of these five chromosomes commonly form ROBs, known as ‘common’ or type I fusions (Page et al., 1996). The two most common fusions are between chromosomes 13 and 14 (75% of ROBs) (Fig. 2B) or between chromosomes 14 and 21 (10% of ROBs) (Therman et al., 1989). Thus, chromosome 14 is involved in 85% of ROBs – an enigmatic observation. Other fusions occur at the level of 1–2%.

Individuals with ROBs are usually phenotypically normal but often suffer from infertility or subfertility and recurrent miscarriages, which can be considered akin to reproductive isolation (Poot and Hochstenbach, 2021). A translocation between chromosomes 14 and 21, when combined with two copies of chromosome 21, will result in a karyotype with the correct number of chromosomes (46), but three copies of chromosome 21, which is effectively a trisomy and causes Down's syndrome (Petersen et al., 1991) (Fig. 2C). In fact, ROBs resulting from the fusion of chromosomes 14 and 21 are the most common cause of age-independent Down's syndrome (Mack and Swisshelm, 2013). Similarly, ROBs resulting from the fusion of chromosomes 13 and 14 contribute to the incidence of Patau syndrome, or trisomy 13 (Patau et al., 1960). Individuals with Patau syndrome often do not survive beyond their early years. ROBs resulting from the fusion of chromosomes 13 and 14 have also been correlated with an increased incidence of breast cancer, but the mechanism is unknown (Schoemaker et al., 2019). Therefore, these translocations can have negative effects on human health.

As mentioned above, ROBs can be a challenge during meiosis; however, at least 50% of ROBs can be inherited (Hook and Cross, 1987), so carriers are not necessarily infertile. In human meiosis, a ROB will synapse with both of its partner chromosomes, forming a trivalent structure (Luciani et al., 1984; Vidal et al., 1982). There are eight possible gamete outcomes, but only two of them will result in an embryo with a normal or balanced karyotype. These outcomes, referred to as the alternate segregation outcome (see Glossary), are the favored segregation pattern based on analysis of sperm and constitute anywhere from 68% to 94% of all segregation products (for a review see Griffin and Ogur, 2023). Studies suggest that male carriers of ROBs produce more euploid embryos than female carriers (Zhang et al., 2019). Interestingly, ROBs resulting from the fusion of chromosomes 13 and 14 have higher rates of alternate segregation than those resulting from the fusion of chromosomes 14 and 21. I speculate that this could be due to insufficiency of crossovers with the normal chromosome 21 because of its small size, combined with the loss of homologous sequences in the ROB. The two smallest chromosomes in the human genome, chromosomes 21 and 22, give rise to 12% of all the abnormal segregations observed in oocytes even though they constitute less than 4% of the genome, and on average have less than a single crossover each per meiosis (Ottolini et al., 2015). Some studies have suggested that carriers of ROBs produce gametes with increased aneuploidy of non-ROBs, referred to as the interchromosomal effect (ICE; see Glossary), although other studies find no evidence for ICE (Griffin and Ogur, 2023). In mice, ROBs increase heterologous chromosome interactions, synapsis and recombination in meiosis, suggesting that they can impact non-ROBs (Vara et al., 2021). As the molecular structures of human ROBs are resolved, they will likely shed light on why some carriers have more positive reproductive outcomes than others, improve predictions for outcomes based on the specific ROB, and enhance preimplantation genetic testing.

## Progress on the sequencing and assembly of human acrocentric chromosomes

The breakpoints for human ROBs have long been mysterious, but the common ROBs may share a common breakpoint at the junction between the two joined chromosomes (Page et al., 1996). Recent advances in long-read sequencing technologies and assembly strategies have allowed for significant insight into the sequences present on acrocentric chromosomes. In 2022, the short arms (p arms) of the five human acrocentric chromosomes, where Robertsonian translocations occur, were fully assembled for the first time in a human genome (assembly CHM13). These assemblies provided the first linear maps for these regions and identified new repeat elements (Nurk et al., 2022; for a recent review see McStay, 2023). Subsequent analysis of these p arms in ∼90 human haplotypes (see Glossary) has led to the astonishing realization that the p arms of the five human acrocentric chromosomes share megabase-sized blocks of sequence homology, including many repetitive DNAs, suggesting frequent and ongoing recombination (Guarracino et al., 2023). The shared homologous regions have been termed pseudohomolog regions (PHRs), akin to the pseudoautosomal regions (PARs) on human X and Y chromosomes that recombine during meiosis (see Glossary). These PHRs fall outside the centromere regions that have low levels of recombination (Langley et al., 2019; Nambiar and Smith, 2016). The discovery of the PHRs demonstrates that the previously unassembled 8% of the genome contains information that will unlock how structural variation occurs in the human genome, and likely in other non-human genomes as well.

**Fig. 3.**
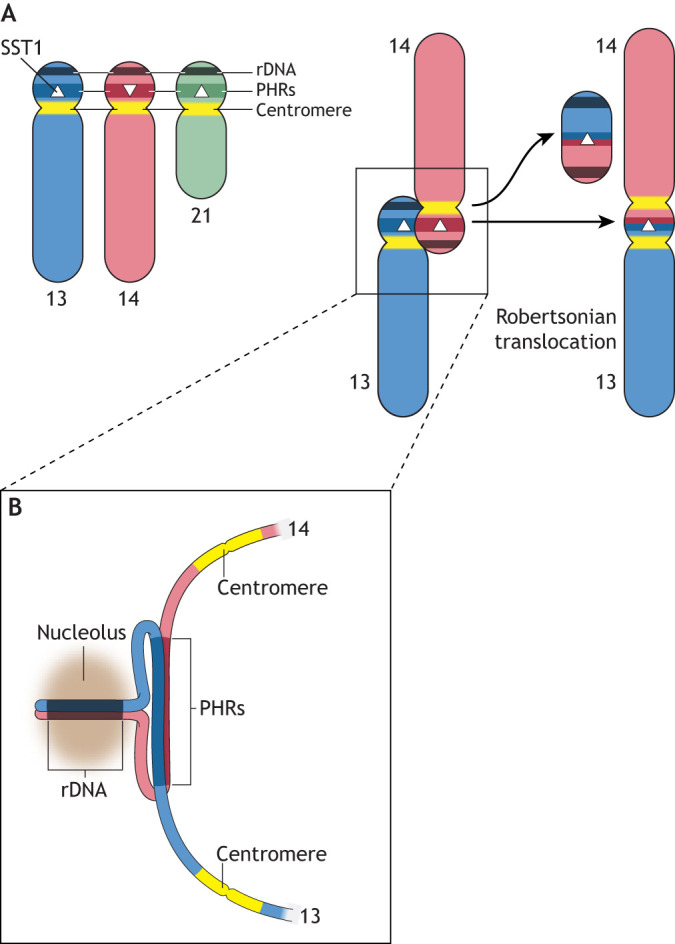
**A model for the formation of human Robertsonian chromosomes.** (A) Chromosomes 13, 14 and 21, with PHRs shown in darker colors. Centromeres are depicted in yellow. Within each PHR is a macrosatellite DNA array called SST1, which is depicted by an arrowhead. The SST1 array contains predicted binding sites for the meiotic protein PRDM9 and is inverted on chromosome 14. A crossover recombination event between chromosome 14 and either chromosome 13 or chromosome 21 will result in the fusion of the two long chromosome arms and the two short (p) arms. The joined p arms, containing part of each PHR, the ribosomal DNA (rDNA) and distal junction sequences, satellite DNAs, and telomeres, are predicted to be lost because they do not have a centromere. The fusion of the long arms will result in a ROB containing SST1 repeats at the breakpoint, flanked by two centromeres, and no ribosomal DNA. (B) This fusion event is facilitated by 3D proximity of the PHRs, which can occur when rDNA arrays from non-homologous chromosomes share the same nucleolus.

Human ROBs tend to form *de novo* between maternal chromosomes, implicating meiotic recombination in oocytes as their main time and place of origin (Bandyopadhyay et al., 2002; Page and Shaffer, 1997; Petersen et al., 1991). The structure of human ROBs has previously been probed using fluorescently labeled bacterial artificial chromosomes (BACs) containing chunks of sequences from these chromosomal regions, to ascertain at a gross level which sequences are retained and which are lost (Jarmuz-Szymczak et al., 2014). This analysis has narrowed down the breakpoint to a large domain on the p arms of the acrocentric chromosomes, likely between the ribosomal DNA and the centromere (Fig. 3). However, without sequence assemblies, it has been difficult to determine the breakpoint with precision.

## Recent insights into the formation of human Robertsonian chromosomes

In a 2023 study, Guarracino et al. used the sequences of previously utilized BACs (Jarmuz-Szymczak et al., 2014), which pre-dated any sequence assemblies of the short arms of human acrocentric chromosomes, and mapped them to the assembled p arms of the CHM13 genome to further pinpoint which sequences are retained and which are lost in common ROBs (Guarracino et al., 2023). This effort led to the realization that the PHRs of the p arms overlap with regions that are retained in ROBs. Careful sequence analysis of the PHRs revealed that a subregion on chromosome 14 is inverted relative to the corresponding regions of chromosomes 13 and 21 (Guarracino et al., 2023), suggesting that if these segments are aligned and a crossover recombination event occurs between chromosome 14 and either chromosome 13 or chromosome 21, this subregion would join the long arms of the chromosomes, as occurs in a ROB. The joined p arms would be lost, since they lack a centromere. This forms the basis for the working model presented in Fig. 3.

One prime candidate for the breakpoint is the SST1 macrosatellite DNA, so named because it bears a restriction site for the enzyme SstI (Tremblay et al., 2010). SST1 tandem repeat arrays (see Glossary) are present on many chromosomes in the human genome (Hoyt et al., 2022) and are also found in primates (Hartley et al., 2021). They are present in the PHRs on chromosomes 13, 14 and 21 (but not in the PHRs of chromosomes 15 or 22), which is consistent with their involvement in homologous recombination to generate the common ROBs. Interestingly, the version of the SST1 repeat on chromosomes 13, 14 and 21 is shorter (∼1.4 kb) compared to the SST1 repeats in the rest of the genome (∼2.4 kb) (Guarracino et al., 2023). It also has predicted binding sites for PRDM9 (Altemose et al., 2017), which is a protein that uses zinc fingers to bind to specific sequences and methylate histone H3 at K4 and K36 (Grey et al., 2018). This facilitates recruitment of the meiotic double-strand break machinery that initiates recombination and creates a hotspot (reviewed in Grey et al., 2018). The results of phylogenetic analysis of SST1 monomers are consistent with ongoing sequence exchange between SST1 arrays on chromosomes 13, 14 and 21 (Guarracino et al., 2023). Furthermore, the SST1 array on chromosome 14 is inverted relative to SST1 arrays on chromosomes 13 and 21 in the haplotypes from multiple independent human genome assemblies, suggesting this is a predominant structural feature that could enable the fusion of the long arm of chromosome 14 with the long arm of either chromosome 13 or chromosome 21. Additional evidence that SST1 is coincident with the breakpoint awaits the complete assembly of human ROBs, but the phylogenetic pattern of SST1 is a smoking gun for a local recombination hotspot.

Although recombination between the PAR regions of the X and Y chromosomes is normal, meiotic recombination between non-homologous chromosomes is not. However, recombination between non-homologous chromosomes, sometimes referred to as non-allelic homologous recombination (see Glossary), is likely how ROBs form in general. Typically, homologous chromosomes are restricted to recombining with each other due to the physical constraints of the synaptonemal complex, a proteinaceous structure that forms between homologs in meiosis (see Glossary) (Page and Hawley, 2004). However, colocalization of the ribosomal DNA arrays from non-homologous chromosomes in the same nucleolus, as has been documented to occur in human oocytes and spermatocytes (Cheng and Naluai-Cecchini, 2004; Stahl et al., 1991), would provide three-dimensional (3D) proximity of the adjacent PHRs to facilitate recombination.

A model that integrates all of this new information has recently been published (Guarracino et al., 2023). In the model, PHRs are brought into proximity by the clustering of the ribosomal DNA arrays from two different chromosomes in the same nucleolus. A local double-strand break occurs by virtue of the predicted PRDM9 binding sites in the SST1 arrays on chromosomes 13, 14 and 21; a break then leads to recombination. Most meiotic double-strand breaks (>90%) resolve as gene conversion, which could occur between any pair of PHRs and generate sequence exchange yielding the homology signatures observed. However, a rarer crossover event between chromosome 14 and either chromosome 13 or chromosome 21 would join the long arms of the chromosomes and, reciprocally, join the p arms. The p arm product is lost, since it has no centromere, while the long arm product is predicted to be dicentric, consistent with previous observations based on imaging (Sullivan and Schwartz, 1995; Sullivan et al., 1994), and would lack ribosomal DNA arrays.

## A working model for how Robertsonian chromosomes form

Given the advances in understanding the formation of common ROBs in the human genome, I elaborate on the aforementioned model and speculate that the formation of ROBs in general relies on three conditions: (1) homology blocks between acro- or telo-centric chromosomes; (2) recombination initiation at or near the homology blocks; and (3) 3D proximity of the homology blocks in germ cells. I speculate that these three criteria need to be satisfied to form ROBs in any genome (Fig. 3). I now consider how each of these criteria might be satisfied more generally, using mouse chromosomes as an example case.

### Homology blocks

Many genomes contain a surfeit of repetitive DNA, with some repeats present on multiple chromosomes. However, some of the largest blocks of repetitive DNA in a genome likely lie in regions that have been incompletely assembled, as was true until recently for the p arms of human acrocentric chromosomes. However, the combination of new sequencing technologies and computational methods will grant access to these regions of genomes. Regarding the remarkable incidence of ROBs in house mouse populations, which can occur between any two telocentric chromosomes, I speculate that the shared centromeric sequences are key. In the *M. m. domesticus* genome, all centromeres consist of minor satellite DNA, which is where the kinetochore is assembled, and major satellite DNA, which is where sister chromatid cohesion occurs (see Glossary) (Guenatri et al., 2004). I speculate that these are the homology blocks that facilitate the formation of ROBs in the mouse genome. The homology blocks involved in the formation of ROBs in other species, such as *Agrodiaetus* butterflies (Lukhtanov et al., 2020, 2005), might be repetitive DNAs that are currently not identified or well annotated.

### Recombination

The event initiating formation of a ROB is likely to be a double-strand break, followed by crossover recombination. Previous studies using trio-based mapping of satellite DNA have ascertained that *de novo* formation of human ROBs occurs primarily during female meiosis (Bandyopadhyay et al., 2002; Page and Shaffer, 1997). This method uses microsatellite variants in the mother, father and child to demonstrate the parental origin of chromosomes. I speculate that there could be several factors that contribute to the higher frequency of ROBs in human female meiosis. Firstly, spindle assembly checkpoints are weaker in female meiosis, so atypical synapsis, recombination and segregation may be better tolerated (Nagaoka et al., 2012). Secondly, there are more crossovers in female meiosis (Broman et al., 1998). Thirdly, sexual dimorphism in recombination hotspots has been documented (see Glossary) (Bherer et al., 2017), although the hotspot landscape in male and female germ cells in the p arms of the acrocentric chromosomes has not been determined. Hotspot information in human female meiosis is limited, particularly in the p arms of the acrocentric chromosomes, but would help in assessing the existence and impact of sexual dimorphism in the formation of ROBs. More research is therefore required to understand the reasons why ROBs arise more frequently in human female meiosis.

In telocentric chromosomes, such as those found in mice, there is evidence for different triggers for the initiation of recombination events that form ROBs (Slijepcevic, 1998). First, the erosion of telomeres, due to a failure in end replication, could trigger the formation of ROBs (Sanchez-Guillen et al., 2015). In telomerase-mutant mice, ROBs form spontaneously after several generations (Blasco et al., 1997). Presumably the lack of telomeric DNA is detected as a double-strand break, and chromosomal fusion occurs. ROBs might also form due to breaks in or near the minor satellite sequence, which serves as part of the centromere (Garagna et al., 1995; Nanda et al., 1995). Because this sequence is shared by all mouse chromosomes, a break in this region could create the opportunity for a crossover event that would fuse two telocentric chromosomes, creating a metacentric chromosome, and the fusion of the two telomeric fragments would be lost. Robertsonian fusions can also occur without the loss of telomeric repeats (Ruiz-Herrera et al., 2008). The mechanism by which a ROB forms, and the precise structure of the fusion chromosome, may result in either a strong or a weak centromere relative to the rest of the karyotype. Variation in the centromeres generated from different events might explain why meiotic drive can favor either the telocentric or the metacentric chromosome.

Recombination events could also be influenced by PRDM9, which contains a histone methyltransferase domain and a hypervariable zinc-finger domain that helps to dictate its DNA binding sites. Many alleles of this gene exist in mammals (Schwartz et al., 2014; Stevison et al., 2016). There is evidence that the landscape of alleles in mice could be related to the incidence of ROBs (Capilla et al., 2014) and hybrid sterility (Davies et al., 2016). How the combinatorial interactions between recombination proteins and DNA sequences influence the formation of ROBs in each genome is an area worthy of research in the future.

### Physical proximity

Crossover recombination between non-homologous chromosomes in meiosis is expected to be a rare event due to the constraints of the synaptonemal complex that forms between homologs (Page and Hawley, 2004). I imagine that for recombination between non-homologous chromosomes to occur, shared homologous sequences are clustered in 3D space to provide the proximity needed for recombination events. In the case of the PHRs, these regions are adjacent to the ribosomal DNA arrays (Guarracino et al., 2023), which can co-cluster in the same nucleolus (Fig. 3). In other species, different types of organizational clustering occur in germ cells. For example, germ cells in many species, including the house mouse, have a structure called a chromocenter, wherein centromeres cluster together (Spangenberg et al., 2021), bringing centromeres and adjacent pericentromeric and telomeric sequences together in 3D space. In the specific case of the house mouse, all centromeres share minor and major satellite sequences, and centromeres cluster, presenting an opportunity to form a ROB if recombination is initiated. Telomere clustering in meiosis, which occurs in many organisms, might also bring sequences together in 3D. In the future, combining fluorescence *in situ* hybridization (FISH) with high-resolution imaging, as well as long-read HiC methods (see Glossary) like PoreC (Li et al., 2022) to capture genome organization in germ cells, will provide new information about chromosomal regions that have physical proximity in meiosis. Whereas previous FISH methods can typically be used to visualize only two or three DNA sequences at a time, new methods to examine many sequences at once in nuclei (Payne et al., 2021) will allow the investigation of chromosome organization on a whole new level.

## Conclusions and future perspectives

One challenge for understanding ROB formation is that it is difficult to obtain sufficient material from germ cells to discover recombination initiation sites, especially for oocytes. Genomics methods that can utilize fewer cells, such as cleavage under targets and tagmentation (CUT&Tag; Kaya-Okur et al., 2020), will help with sample scarcity. Methods based on long-read sequencing, such as directed methylation with long-read sequencing (DiMeLo seq; Altemose et al., 2022), will advance efforts for mapping of recombination initiation sites within repetitive DNA. Moreover, existing meiotic recombination hotspot data, such as those generated for recombination proteins like DMC1 and SPO11, can be remapped as new complete genome assemblies become available for a given species (Lange et al., 2016; Pratto et al., 2014). Super-resolution imaging efforts, which involve single-cell data acquisition and therefore require fewer cells, will also provide advances. For example, immunoFISH (Yu and Potapova, 2022) in germ cells, using a DNA FISH probe to a suspected recombination-initiating sequence combined with immunofluorescence for protein machinery associated with double-strand breaks, might be helpful in discovering recombination hotspots and provides an opportunity to monitor the frequency of break sites at the single-cell level.

In summary, recent advances in sequencing and assembly methods have suggested how the common ROBs occur in the human genome. However, the meiotic recombination events leading to the formation of rare ROBs remain elusive. Based on advances in understanding common human ROBs, we can begin to make inferences about how these translocations occur more broadly in nature. We are now at a point in time when advances in sequencing technology and imaging-based visualization of chromosomes can provide answers to how these translocations occur in each genome. Given the recent explosion in long-read sequencing and assembly technologies, the structure of ROBs in many species should be resolvable in the next 5–10 years.

It is important to continue to understand these translocations because they impact fertility, karyotype evolution and speciation. Understanding the breakpoints and mechanisms involved in ROB formation will advance models of chromosome evolution and highlight roles for specific repetitive DNAs. Resolving the structure of the human translocations might facilitate better preimplantation genetic testing for carriers, enhance our understanding of the risks and benefits associated with specific translocations, and lead to improved counseling and fertility outcomes.
